# The status of binocular visual functions among Taiwan high-tech industry engineers and its correlation with computer vision symptom

**DOI:** 10.1038/s41598-024-51314-1

**Published:** 2024-01-08

**Authors:** Lung-Hui Tsai, Bo-Yu Chen, Kuo-Chen Su, Ching-Ying Cheng

**Affiliations:** 1 Department of Optometry, Chung Shan Medical University, Taichung, 402 Taiwan; 2 Department of Ophthalmology, Chung Shan Medical University Hospital, Taichung, 402 Taiwan

**Keywords:** Health care, Health occupations

## Abstract

To analyze the status of binocular visual functions, the relationship between binocular visual function and computer vision-related symptoms in the high-tech industry group. The study sample was comprised of 33 participants aged between 20 and 40 years of age. After completing basic information and the Computer Vision Symptom Scale (CVSS-17) questionnaire, the participants underwent a comprehensive examination of binocular visual function. All data were statistically analyzed with SPSS V26.0 software. The value of the binocular vision function of the Taiwan high-tech industry group was significantly different compared with the Scheiman and Morgan standard value. Study subjects were generally found to exhibit larger exophoric at distance, which in turn might lead to a lower ability to maintain binocular fusion to a single image, or recover from fusional disruption at distance. Subjects also experienced accommodation and convergence problems at near at the same time. Age, gender, and refractive errors had no significant impact on CVSS-17 scores, only the duration of computer usage showed a significant effect, particularly for internal symptom factor (ISF) dimensions. In addition, the interaction between the ISF and external symptom factor resulted in more severe visual symptoms. Long-term use of electronic devices may lead to an imbalance in binocular vision function, thereby increasing or exacerbating visual symptoms. If the use of electronic devices is an unchangeable trend, interventions in prescription, visual training or the visual design of electronic products become worthwhile topics for development.

## Introduction

Taiwan's high-tech industry occupies a pivotal position in the global market, and consequently, the proportion of people engaged in the high-tech industry is also remarkably high. The internet, computers, and other related electronic products are practically omnipresent in every corner of Taiwan^1,2^; however, research has indicated that the prolonged use of electronic devices can result in symptoms of discomfort, including eye soreness, redness, pain, dryness, burning sensation, and blurred vision^3,4^, which are collectively referred to as Computer Vision Syndrome (CVS).

From a clinical perspective, CVS appears to have a significant correlation with binocular vision^5,6^, which is closely related to visual performance in daily life^7,8^. Patients with binocular vision abnormalities typically show many ocular physiological and psychological responses in a clinical setting, such as blurred vision, headache, eye strain or discomfort, intermittent diplopia, inattention, eye rubbing, excessive blinking, and photophobia^9–14^. For example, minor esophoria and exophoria usually do not cause severe discomfort for patients in a clinical setting. However, when individuals engage in near reading, both eyes must move inward simultaneously. In a condition where equal convergence demands are met, individuals with a basic exophoric eye position require more effort to achieve a clear and stable visual effect^15^.

Patients with such conditions often complain of eye fatigue, blurred vision, and diplopia during reading or near work. Those with more severe symptoms or higher visual demands may even experience difficulty concentrating or headaches. The symptoms of binocular vision dysfunction above are very similar to CVS and dry eye^5,6^; thus, the mutual influence among CVS, dry eye, and binocular visual functions has also made the diagnostic process more complex in many clinical cases^16–20^. High-tech engineers may experience more severe dry eye and CVS symptoms due to prolonged and specific computer use. However, no research has demonstrated a relationship between dry eye and CVS in high-tech industry professionals, specifically regarding binocular vision. This lack of evidence serves as the primary motivation for conducting this study.

The Computer Vision Symptom Scale questionnarie (CVSS-17) quantifies the visual performance of professionals who spend prolonged periods using computers^21–23^. This questionnaire analyzes the potential effects of prolonged computer use and classifies them based on the frequency and severity of various symptoms, thereby determining the overall severity of different symptoms. The participants in this study were all professionals in the high-tech industry who commonly use desktop or laptop computers for work during their working hours. The purpose of this study was to investigate the statues of binocular visual functions and the relationship between binocular visual function and visual symptoms in high-tech industry engineers.

## Materials and methods

### Study design

The research was a prospective study, which was approved by the Institutional Review Board of the First Human Experimentation Committee of Chung Shan Medical University Affiliated Hospital. All participating researchers possessed good clinical practice education and training certificates for human trials and strictly adhered to the ethical principles of the Helsinki Declaration. The study was conducted at the specialized optometry laboratory of the Department of Optometry at Chung Shan Medical University.

The external conditions of the optometry room remained constant, avoiding potential interference factors such as differences in brightness between different rooms, the distance of vision charts, and subjective variations among examiners, to maintain the accuracy of the test results. After receiving an explanation from the researchers and signing the informed consent form, participants were required to complete the CVSS-17 questionnaire as a preliminary step. Subsequently, data regarding visual acuity, refractive errors, and binocular visual function were also collected for inclusive or exclusive considerations. The binocular visual examination included phoria at near and distance, convergence, divergence, and accommodative abilities. All examination data were compared with Morgan and Scheiman’s Optometric Extension Program^24,25^ which has been well established since 1944.

## Research subjects

To prevent presbyopia, serious dry eyes, and contact lenses wear(since the use of contact lenses has a different accommodation demand than glasses, and this issue itself can cause dry eyes in people using contact lenses) from affecting the analysis results of binocular visual functions, the study recruited adults aged 20 to 40 years old and had been employed as senior engineers in high-tech companies over 1 years. High-tech industry in this study adopted the definitions provided by the United Nations, the European Union, and developed countries including the United States, Japan, and representative international organizations^26^. After providing an explanation, 51 healthy adults consent to participant, The exclusion criteria were as follows: individuals with refractive errors of sphere ≤ − 8.00 D or > + 1.00 D; astigmatism ≤  − 1.00 D; long-term use of contact lenses, best-corrected visual acuity below 1.0 in either eye, eye-related diseases(including dry eye), previous eye or brain surgery, psychological disorders, pregnancy, amblyopia, or physiological conditions affecting the immune system, or metabolism. Of these, 33 subjects were enrolled after screening, 18 were subsequently excluded: 7 subjects had refractive errors that did not meet the criteria, 1 had refractive surgery, 1 were pregnancy, 1 had immune system disease, 6 were long-term contact lenses user, 2 did not cooperate with the follow-up schedule. Finally, 33 subjects with a mean age of 28.82 ± 4.66 years participated. The Shapiro–Wilk test revealed that the participants’ spherical equivalent power data were under normally distributed (right eye: W = 0.949, *p* = 0.121; left eye: W = 0.961, *p* = 0.268). The participants’ characteristics are summarized in Table 1.

**Table 1:** Basic information of the participants.

	Group1	Group2	Maximum	Minimum	mean	SD
Gender						
(Group1. Male/Group 2. Female)	15(45.5%)	18(54.5%)				
Age						
(Group 1. < 30 / Group 2. ≥ 30)	17(51.5%)	16(48.5%)	36	21	28.82	4.66
Refractive error-OD						
(Group 1. SE < −5.00D / Group 2. ≥ −5.00D)	13(39.4%)	20(60.6%)	− 7.25	− 0.25	− 4.68	3.04
Refractive error-OS						
(Group 1. SE < −5.00D / Group 2. ≥ −5.00D)	12(36.4%)	21(63.6%)	− 7.75	0	− 4.43	2.88
Computer time						
(Group 1. < 8 h / Group 2. ≥ 8 h)	15(45.5%)	18(54.5%)	15	4	9.16	3.19

The sample size of this study was determined using G*Power analysis, with an effect size of f = 0.8, α = 0.05, power (1 − β) = 0.95, and number of groups = 1. The calculated results of the total sample size were 27. The number of participants who completed the study (33) exceeded the sample size required (27); the power appeared to be adequate after recalculation and adjustment (effect size f = 0.8, α = 0.05, power (1 − β) = 0.878).

## Research materials

The View-M Digital Visual Acuity Chart (Quan Chin Industrial Co., Taiwan) and Near Visual Acuity Chart (Brighten Optix Co., Taiwan) were used as visual assessment tools. The Nidek AR 800 Autorefractor (Nidek, Seoul, Korea) was used for computerized refractive errors, followed by retinoscopy for confirmation. Subjective refraction was conducted using the TOPCON VT-10 phoropter (Topcon, Tokyo, Japan). The Von Graefe phoria and Maddox rod phoria test were performed to evaluate distance and near phoria (DLP and NLP), and distance and near fusional convergence and divergence (DBI, DBO, NBI, and NBO). The Royal Air Force rule (Bernell, Mishawaka, USA)was used to measure the near point of convergence (NPC) and amplitude of accommodation (AA). The gradient AC/A ratio was calculated by simultaneously placing + 1.00 D and − 1.00 D lenses over both eyes and measuring the change by each lens in the near eye position. The Random Dot Stereo Test (Bernell, Mishawaka, USA)was utilized to assess stereopsis, and monocular and binocular accommodative facilities were measured using ± 2.00 D flip lenses.

The CVSS17 questionnaire contains 17 items with different rating scales. Two items have two response categories, eleven items have three response categories, and four items have four response categories. The questionnaire gives information about 15 different symptoms, considering the severity and frequency of symptoms. The high internal consistency (Cronbach’s α = 0.92) of the Spanish version makes it useful for comparisons between groups and for clinical applications. This questionnaire was designed for examining CVS symptom type and severity, and can divide participants by their CVS severity score. Factor analysis divided the questionnaire into two dimensions, the internal symptom factor (ISF) and external symptom factor (ESF). The ESF pattern comprises burning, irritation, tearing, and dryness located in the front and bottom of the eye. ESF is caused by holding the eyelid open, glare, up gaze, small font, and flickering. ESF seems highly related to dry-eye symptoms. The ISF pattern comprises ache, strain, and headache located behind the eyes. ISF is caused by the close viewing distance, lens flipper, and mixed astigmatism conditions and is likely related to accommodative and vergence stress^27^.

### Data analysis and statistical analysis

The statistical methods used in this study were the one sample t-test, one-way ANOVA, linear and Logistic regression, two-way ANOVA, and chi-square test. These analyses were conducted using IBM SPSS Statistics v.26 software (IBM Corp., Armonk, NY). A *p*-value < 0.05 was considered statistically significant.

### Ethical approval

The study was conducted according to the guidelines of the Declaration of Helsinki and approved by the Ethics Committee of Chung Shan Medical University Hospital (Taichung, Taiwan) (approval number: CS19110). Informed written consent was obtained from all individual participants included in the study.

### Informed consent

Patients signed informed written consent regarding the publication of their data or photographs.

## Results

The study collected valid data from 33 participants, including 15 males and 18 females, with an average age of 28.82 ± 4.66 years. The average equivalent spherical power was: right eye − 4.68 ± 3.04 D, and left eye − 4.43 ± 2.88 D. There was no significant difference in refractive error between gender, age, as well as between the left and right eyes. Therefore, there was no need for specific analysis based on gender, age or the left–right eye distinction.

### The status of binocular vision among high-tech industry engineers

#### Binocular visual functions and Morgan’s Norm comparison at distance

The values of Taiwan high-tech industry engineers were generally found to be worse than the standard values (Table 2). The MR DLP (t = − 1.264, *p* = 0.216), MR NLP (t = − 1.890, *p* = 0.068), DBO Break (t = 0.024, *p* = 0.981), DBO Recovery (t = − 0.616, *p* = 0.542), and one sample t-test revealed that subjects showed larger exophoric status at distance (DLP: t = − 4.548, *p* < 0.001), which in turn affected the ability to diverge (DBI-Break: t = 6.616, *p* < 0.001; DBI-Recovery: t = 3.870, *p* = 0.001), and converge (DBO-Blur: t = 2.973, *p* = 0.006). Subjects might have a lower ability to maintain binocular fusion of a single image, or recover from fusional disruption at distance. Similar results were observed in the analysis of the t-test for distance binocular visual functions when compared to the Taiwan norm.

**Table 2:** Binocular visual function comparison.

Binocular vision	Present findings (means ± SD)	Morgan’s norm (means ± SD)	t-test	*p*-Value	Taiwan norm (means ± SD)	t-test	*p*-value
DLP	4 exo ± 3.27	1 exo ± 2	− 4.548	**0.000**	1.56 exo ± 3.40	− 3.565	**0.001**
NLP	7 exo ± 6.82	3 exo ± 3	− 3.509	**0.001**	6.01 exo ± 6.92	− 0.974	0.337
DLP (MR)	2.3 exo ± 5.9	1 exo ± 2	− 1.264	0.216	–	–	–
NLP (MR)	5 exo ± 6.35	3 exo ± 3	− 1.890	0.068	–	–	–
DBI Break	12.58 ± 4.8	7 ± 3	6.616	**0.000**	11.01 ± 3.67	1.858	0.072
DBI Recovery	6 ± 2.96	4 ± 2	3.870	**0.001**	4.51 ± 2.62	2.883	**0.007**
DBO Blur	11.36 ± 4.6	9 ± 4	2.973	**0.006**	6.46 ± 7.16	6.167	**0.000**
DBO Break	19 ± 7.3	19 ± 8	.024	0.981	17.34 ± 7.87	1.324	0.195
DBO Recovery	9.33 ± 6.2	10 ± 4	− .616	0.542	6.54 ± 4.34	2.583	**0.015**
NBI Blur	14.9 ± 5.7	13 ± 4	1.908	0.065	6.74 ± 7.10	8.166	**0.000**
NBI Break	25.5 ± 5.5	21 ± 4	4.715	**0.000**	18.50 ± 6.58	7.326	**0.000**
NBI Recovery	14.4 ± 3.49	13 ± 5	2.343	**0.025**	11.51 ± 5.70	4.795	**0.000**
NBO Blur	13.78 ± 6.04	17 ± 5	− 3.052	**0.005**	4.27 ± 7.20	9.044	**0.000**
NBO Break	18.4 ± 6	21 ± 6	− 2.426	**0.021**	16.05 ± 7.00	2.237	**0.032**
NBO Recovery	8.9 ± 3.7	11 ± 7	− 3.237	**0.003**	7.39 ± 5.58	2.351	**0.025**
Gradient AC/A (plus)	1.9545 ± 3.15	4 ± 2	− 3.726	**0.001**	2.35 ± 5.21	− 0.720	0.476
Gradient AC/A (minus)	2.8182 ± 3.19	4 ± 2	− 2.126	**0.041**	2.85 ± 4.54	− 0.057	0.955
NPC-break	7.5 ± 1.97	5 ± 2.5	14.533	**0.000**	6.17 ± 2.70	3.833	**0.001**
NRA	1.674 ± 6.04	2.00 ± 0.50	− 5.253	**0.000**	1.71 ± 0.61	− 0.326	0.746
PRA	− 1.57 ± 0.68	− 2.37 ± 1.00	6.748	**0000**	− 2.04 ± 1.28	3.971	**0.000**
MAF	10.12 ± 2	11 ± 5	− 2.490	**0.018**	10.07 ± 5.08	0.145	0.886
BAF	7.72 ± 1.7	10 ± 5	− 7.596	**0.000**	10.66 ± 4.15	− 9.801	**0.000**
NPA (OD)^#^	5.878 ± 1.33	8.3939	− 6.909	**0.000**	15.16 ± 4.73	− 39.812	**0.000**
NPA (OS)^#^	5.9773 ± 1.3	8.3939	− 6.719	**0.000**			
Stereopsis	60’ ± 35.5	30’	4.853	**0.000**	–	–	–

Significant values are in [bold].

^#^18-(age/3)

DLP, distance lateral phoria; NLP, Near lateral phoria; MR, Maddox rod test; NPC, near point of convergence; NRA, negative relative accommodation; PRA, positive relative accommodation; MAF, monocular-accommodative facility, BAF, binocular-accommodative facility, AC/A, accommodative convergence/accommodation; NBI, near base in; DBI, distance base in; NBO, near base out; DBO, distance base out; exo, exo-phoria.

#### Binocular visual functions and Morgan’s Norm comparison at near

Subjects also showed larger exophoric status at near (NLP: t = − 3.509, *p* = 0.001), and the ability of diverge to maintain binocular fusion was better than expected (NBI-Blur: t = 1.908, *p* = 0.065; NBI-Break: t = 4.715, *p* < 0.001; NBI-Recovery: t = 2.343, *p* = 0.025), whereas the ability to converge was worse (NBO-Blur: t = − 3.052, *p* = 0.005; NBO-Break: t = − 2.426, *p* = 0.021; NBO-Recovery: t = − 3.237, *p* = 0.003). Furthermore, NPA (t = − 6.909, *p* = 0.000), NPC (t = 14.533, *p* = 0.000), stereopsis (t = 4.853, *p* = 0.000), AC/A (plus) (t = − 3.726, *p* = 0.001), AC/A (minus) (t = − 2.126, *p* = 0.041), NRA (t = − 5.253, *p* = 0.000), positive relative accommodation (PRA) (t = 6.748, *p* = 0.000), MAF (t = − 2.490, *p* = 0.018), and BAF (t = − 7.596, *p* = 0.000) all indicated a lower ability in this study.

### The scores of CVSS-17 questionnaires among high-tech industry engineers

**Figure 1:**
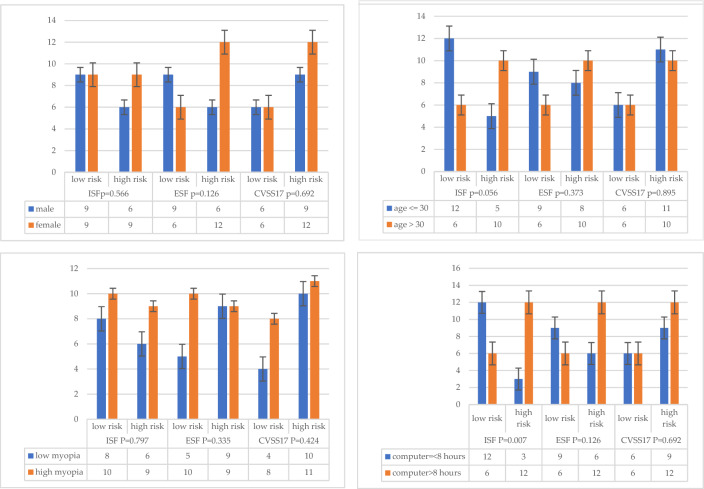
Gender, age, myopia, and computer use time influence on the CVSS17, ISF, and ESF.

**Figure 2:**
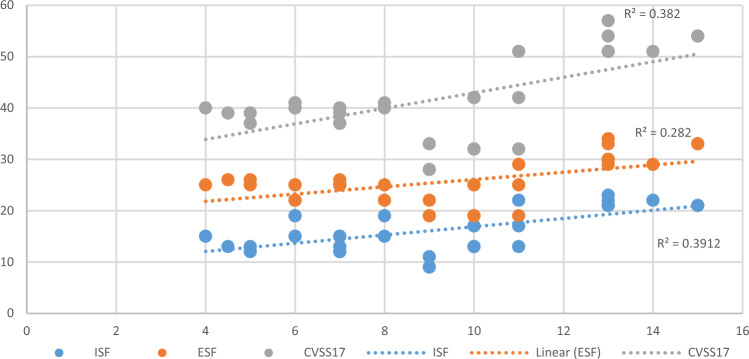
Linear regression between computer use time and the ISF, ESF, CVSS17.

The CVSS-17 questionnaire (Fig. 1) was divided into two dimensions (ISF and ESF) and two levels of performance (low risk and high risk). Although female, elder group (≥ 30 years of age), and high myopia (≤ − 5.00D) had a higher risk in ISF (gender: χ^2^ = 0.330, *p* = 0.566; age: χ^2^ = 3.640, *p* = 0.056; refraction: χ^2^ = 0.066, *p* = 0.797), ESF (gender: χ^2^ = 2.347, *p* = 0.126; age: χ^2^ = 0.793, *p* = 0.373; refraction: χ^2^ = 0.930, *p* = 0.335), and CVSS17 total score (gender: χ^2^ = 0.157, *p* = 0.692; age: χ^2^ = 0.017, *p* = 0. 895; refraction: χ^2^ = 0.638, *p* = 0.424), there was no statistically significant difference by chi-square analysis. Only the daily computer use time had a significant impact on the CVSS17 scores, which had a statistically significant difference in ISF dimensions (χ^2^ = 7.187, *p* = 0.007), indicating that the longer the computer use time, the greater impact on internal symptoms, such as headache or eyestrain. Analyzing the duration of computer use time to predict the questionnaire scores can explain up to 28–39% of the variance (ISF: 39.12%, ESF: 28.20%, CVSS17: 38.20%, Fig. 2).

### Binocular visual functions and CVSS-17 risk group

The CVSS-17 questionnaire contains ISF and ESF dimensions, we conducted a two-factor analysis of variance on binocular visual functions and the CVSS-17, ISF, and ESF.

#### Binocular visual functions and CVSS-17 total score

**Figure 3:**
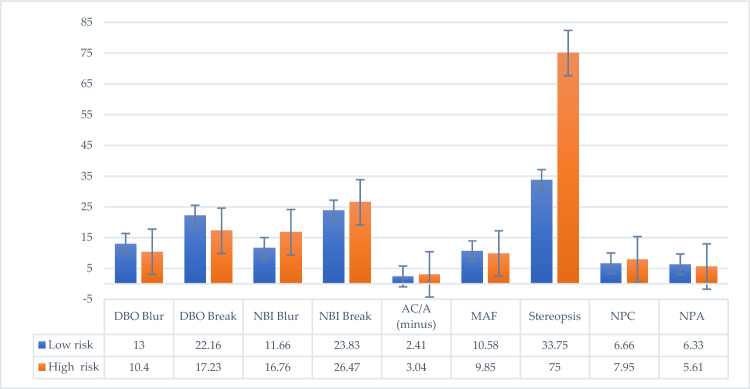
CVSS-17 and binocular visual function.

The results of two-way ANOVA analysis(Table 3) showed that DBO Blur (F = 3.716, *p* = 0.022), DBO Break (F = 3.300, *p* = 0.034), NBI Blur (F = 5.803, *p* = 0.003), NBI Break (F = 2.959, *p* = 0.049), ACA (−) (F = 3.365, *p* = 0.032), MAF (F = 4.278, *p* = 0.013), stereopsis (F = 5.096, *p* = 0.006), NPC (F = 3.341, **p** = 0.033), and NPA (F = 3.623, *p* = 0.025) between the low risk and high-risk groups indicated that the high-risk group of computer vision exhibited lower fusional ability of the convergence and divergence systems when seeing at far, and that the divergence and accommodative system also had a lower ability at near(Fig. 3).

**Table 3:** Two-way ANOVA analysis between CVSS17, ISF, ESF, and binocular visual functions.

Binocular vision	CVSS17 group		ISF group		ESF group		ISF * ESF
	Low-risk group: N = 12		Low-risk group: N = 15		Low-risk group: N = 18		
	High-risk group: N = 21		High-risk group: N = 18		High-risk group: N = 15		
	Risk	ANOVA	Risk	ANOVA	Risk	ANOVA	ANOVA
DLP	L -2.41 ± 1.16	F = 1.962 *p* = 0.142	L -2.41 ± 1.01	F = 3.629 *p* = 0.067	L -2.96 ± 0.99	F = 0.007 *p* = 0.936	F = 0.325 *p* = 0.573
	H -4.26 ± 3.88		H -5 ± 4.39		H -4.11 ± 4.32		
DBI break	L 11.66 ± 1.87	F = 0.556 *p* = 0.648	L 12.7 ± 3.82	F = 0.225 *p* = 0.639	L 12.8 ± 4.19	F = 0.034 *p* = 0.854	F = 1.585 *p* = 0.218
	H 13.09 ± 5.89		H 12.3 ± 5.97		H 12.3 ± 5.43		
DBI recovery	L 5.75 ± 1.35	F = 1.347 *p* = 0.278	L 6.16 ± 2.2	F = 1.098 *p* = 0.303	L 5.80 ± 2.65	F = 1.098 *p* = 0.303	F = 3.559 *p* = 0.069
	H 6.14 ± 3.6		H 5.8 ± 3.76		H 6.16 ± 3.27		
DBO blur	L 13 ± 3.13	**F = 3.716** ***p* = 0.022**	L 13 ± 4.86	**F = 6.906** ***p* = 0.014**	L 12.4 ± 6.05	F = 0.141 *p* = 0.710	**F = 4.741** ***p* = 0.038**
	H 10.4 ± 5.04		H 9.4 ± 3.37		H 10.5 ± 2.7		
DBO break	L 22.16 ± 6.17	**F = 3.300** ***p* = 0.034**	L 20.4 ± 6.94	**F = 6.805** ***p* = 0.014**	L 17.2 ± 8.74	**F = 7.129** ***p* = 0.012**	F = 2.646 *p* = 0.115
	H 17.23 ± 7.46		H 17.3 ± 7.65		H 20.5 ± 5.73		
DBO recovery	L 10.33 ± 3.7	F = 1.276 *p* = 0.301	L 9.33 ± 4.33	F = 0.638 *p* = 0.431	L 8.53 ± 5.3	F = 1.435 *p* = 0.241	F = 3.229 *p* = 0.083
	H 8.76 ± 7.3		H 9.33 ± 8		H 10 ± 6.96		
NLP	L -6.83 ± 8.46	F = 2.001 *p* = 0.136	L -4.69 ± 7.76	F = 3.211 *p* = 0.084	L -5.16 ± 4.95	F = 0.291 *p* = 0.593	F = 0.000 *p* = 0.994
	H -7.35 ± 5.91		H -10.13 ± 3.98		H -8.8 ± 7.8		
NBI blur	L 11.66 ± 3.7	**F = 5.803** ***p* = 0.003**	L 15.4 ± 6.78	F = 0.309 *p* = 0.582	L 18.13 ± 5.42	**F = 9.224** ***p* = 0.005**	F = 3.706 *p* = 0.064
	H 16.76 ± 5.94		H 14.2 ± 4.33		H 12.2 ± 4.59		
NBI break	L 23.83 ± 5.99	**F = 2.959** ***p* = 0.049**	L 26 ± 5.86	F = 1.278 *p* = 0.268	L 26 ± 5.85	F = 0.235 *p* = 0.632	**F = 8.450** ***p* = 0.007**
	H 26.47 ± 5.09		H 24.93 ± 5.17		H 25.1 ± 5.32		
NBI recovery	L 13.16 ± 2.2	F = 0.626 *p* = 0.604	L 14.2 ± 3.07	F = 0.003 *p* = 0.955	L 14.2 ± 3.51	F = 0.162 *p* = 0.691	F = 1.745 *p* = 0.197
	H 15.14 ± 3.91		H 14.6 ± 4.02		H 14.5 ± 3.56		
NBO blur	L 13.83 ± 3.35	F = 2.574 *p* = 0.073	L 15.4 ± 6.98	F = 0.851 *p* = 0.364	L 16.5 ± 6.98	F = 2.709 *p* = 0.111	F = 0.662 *p* = 0.422
	H 13.76 ± 7.23		H 11.8 ± 4.05		H 11.5 ± 4.04		
NBO break	L 17.0 ± 1.8	F = 0.592 *p* = 0.625	L 18.6 ± 6.57	F = 0.004 *p* = 0.948	L 19.4 ± 6.98	F = 0.349 *p* = 0.559	F = 0.970 *p* = 0.333
	H 19.23 ± 7.46		H 18.13 ± 5.68		H 17.5 ± 5.29		
NBO recovery	L 8.5 ± 3.42	F = 2.778 *p* = 0.059	L 9.66 ± 4.4	F = 2.206 *p* = 0.148	L 9.73 ± 4.65	F = 0.004 *p* = 0.953	**F = 5.933** ***p* = 0.021**
	H 9.14 ± 3.92		H 8 ± 2.5		H 8.22 ± 2.64		
NRA	L 2.0 ± 0.74	F = 2.699 *p* = 0.064	L 1.83 ± 0.66	F = 0.122 *p* = 0.730	L 1.98 ± 0.60	**F = 5.148** ***p* = 0.031**	F = 0.190 *p* = 0.666
	H 1.48 ± 0.47		H 1.48 ± 0.53		H 1.41 ± 0.54		
PRA	L -2.06 ± 0.73	F = 2.312 *p* = 0.097	L -1.79 ± 0.72	**F = 4.243** ***p*** = **0.048**	L -1.73 ± 0.79	F = 0.000 *p* = 1.000	F = 2.034 *p* = 0.165
	H -1.28 ± 0.46		H -1.3 ± 0.53		H -1.43 ± 0.55		
AC/A (plus)	L 0.87 ± 2.41	F = 2.295 *p* = 0.099	L 2.25 ± 2.85	F = 0.381 *p* = 0.542	L 3.4 ± 1.81	**F = 6.090** ***p* = 0.020**	F = 0.000 *p* = 1.000
	H 2.57 ± 3.4		H 1.6 ± 3.54		H 0.75 ± 3.55		
AC/A (minus)	L 2.41 ± 3.61	**F = 3.365** ***p* = 0.032**	L 1.69 ± 3.49	**F = 8.595** ***p* = 0.007**	L 3.16 ± 3.13	F = 3.505 *p* = 0.071	F = 0.080 *p* = 0.780
	H 3.04 ± 2.9		H 4.16 ± 2.21		H 2.52 ± 3.3		
MAF	L 10.58 ± 1.31	**F = 4.278** ***p* = 0.013**	L 10.8 ± 1.42	F = 0.848 *p* = 0.365	L 11.2 ± 1.27	**F = 6.415** ***p*** = **0.017**	F = 0.212 *p* = 0.649
	H 9.85 ± 2.32		H 9.26 ± 2.34		H 9.16 ± 2.06		
BAF	L 8 ± 1.8	F = 2.796 *p* = 0.058	L 8.11 ± 1.64	F = 0.066 *p* = 0.799	L 8.6 ± 1.29	**F = 5.339** ***p*** = **0.028**	F = 0.066 *p* = 0.799
	H 7.57 ± 1.69		H 7.26 ± 1.75		H 7 ± 1.71		
Stereopsis	L 33.75 ± 11.3	**F = 5.096** ***p* = 0.006**	L 49.7 ± 29.37	F = 0.413 *p* = 0.526	L 49.6 ± 32.3	F = 2.745 *p* = 0.108	**F = 9.889** ***p* = 0.004**
	H 75 ± 36		H 72.339.1		H 68.6 ± 36.5		
NPC	L 6.66 ± 2.49	**F = 3.341** ***p*** = **0.033**	L 7.33 ± 2.37	F = 1.060 *p* = 0.312	L 6.46 ± 2.16	**F = 9.540** ***p*** = **0.004**	F = 0.118 *p* = 0.734
	H 7.95 ± 1.46		H 7.66 ± 1.39		H 8.3 ± 1.32		
NPA	L 6.33 ± 1.55	**F = 3.623** ***p* = 0.025**	L 6.25 ± 1.49	**F = 6.842** ***p*** = **0.014**	L 5.95 ± 1.57	F = 1.683 *p* = 0.205	**F = 6.622** ***p* = 0.015**
	H 5.61 ± 1.16		H 5.43 ± 1.00		H 5.81 ± 1.14		

Significant values are in [bold].

#### Binocular visual functions and CVSS-17 ISF

The results of two-way ANOVA analysis (Table 3) showed that DBO Blur (F = 6.906, *p* = 0.014), DBO Break (F = 6.805, **p** = 0.014), PRA (F = 4.243, *p* = 0.048), ACA (−) (F = 8.595, *p* = 0.007), and NPA (F = 6.842, *p* = 0.014) between the ISF low-risk and high-risk groups indicated that the high-risk group of computer vision exhibited a lower AA, lower PRA, and NPC.

**Figure 4:**
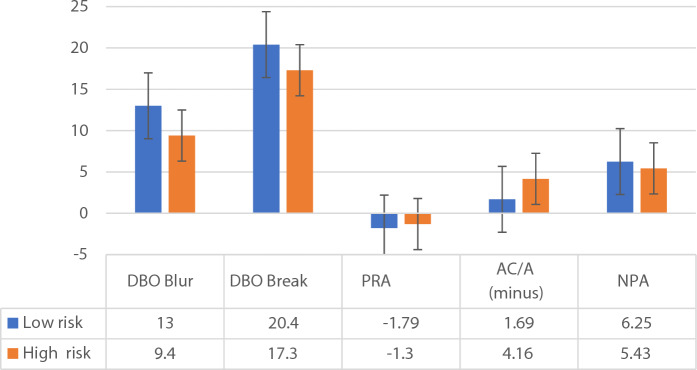
CVSS17 ISF and binocular visual function.

Although there was no significant difference in distance phoria between the two groups, the high-risk group still had a higher exophoria. This, in turn, affected their ability to maintain a single fused image in the distance. Apart from poor monocular accommodation, the difference in PRA indicated binocular accommodative dysfunction was also present, inducing convergency problems. The high-risk group in ISF may frequently experience blurred vision at near, or blurred vision while switching between near and distance, double vision, strabismus, inability to sustain prolonged reading, or eye soreness after extended near work (Fig. 4).

#### Binocular visual functions and CVSS-17 ESF

**Figure 5:**
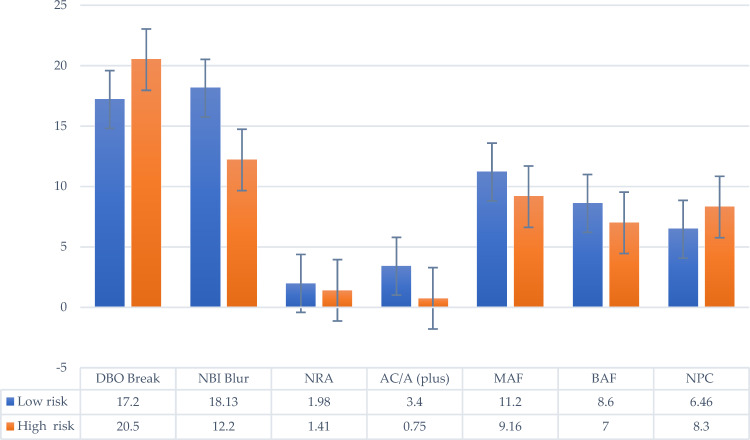
CVSS17 ESF and binocular visual function.

The results of two-way ANOVA analysis(Table 3) showed that DBO Break (F = 7.129, *p* = 0.012), NBI Blur (F = 9.224, *p* = 0.005), NRA (F = 5.148, *p* = 0.031), ACA (+) (F = 6.090, *p* = 0.020), MAF (F = 6.415, *p* = 0.017), BAF (F = 5.339, *p* = 0.028), and NPC (F = 9.540, *p* = 0.004) between the ESF low risk and high-risk groups indicated the high-risk group of computer vision exhibited excessive divergence when looking at distant and near objects, and experienced accommodation and convergence problems at the same time. Patients may feel heaviness, stinging, dryness, and experience redness or burning sensation after long-term computer use (Fig. 5).

#### ISF and ESF interactions on binocular visual functions

Two-way ANOVA analysis showed the variables that exhibited an interaction effect in the performance of the ISF and ESF questionnaires on binocular vision (Table 3) were as follows: DBO Blur (F = 4.741, *p* = 0.038), NBI Break (F = 8.450, *p* = 0.007), NBO Recovery (F = 5.933, *p* = 0.021), stereopsis (F = 9.889, *p* = 0.004), and NPA (F = 6.622, *p* = 0.015). Under the interactive conditions of the ISF and ESF, the eye or physiological symptoms may worsen leading to visual problems such as photophobia, tearing, and a sensation of tightness in the eyes.

The results of logistic regression analysis (Table 4) put emphasis on the accommodation system again which indicated that NRA, PRA, NPA, and stereopsis can effectively predict the risk of CVS, so it is important to include accommodative-related tests as part of routine vision examinations. In addition, phoria and accommodative function can significantly predict the risk of ISF, while vergence and accommodative function can significantly predict the risk of ESF. In clinical practice, it is important for eye care and vision professionals to determine the examination items for binocular vision based on the patient's complaints. This requires specific training in the field of ophthalmology and optometry. However, due to the limited number of participants included in the study, the predictive ability of logistic regression might be insufficient. Therefore, the results analysis should be considered as reference only, and future validation should be conducted using more quantitative data.

**Table 4:** Logistic regression analysis for predicting CVSS17 and binocular visual function.

Binocular vision	ISF	ESF	CVSS17	Binocular vision	ISF	ESF	CVSS17
DLP	**Exp(B) = 0.744**	Exp(B) = 0.892	Exp(B) = 0.824	NBO Break	Exp(B) = 0.985	Exp(B) = 0.948	Exp(B) = 1.071
	***p* = 0.035**	*p* = 0.317	*p* = 0.129		*p* = 0.800	*p* = 0.369	*p* = 0.314
DBI Break	Exp(B) = 0.981	Exp(B) = 0.982	Exp(B) = 1.068	NBO Recovery	Exp(B) = 0.873	Exp(B) = 0.889	Exp(B) = 1.051
	*p* = 0.790	*p* = 0.805	*p* = 0.412		*p* = 0.205	*p* = 0.249	*p* = 0.628
DBI Recovery	Exp(B) = 0.958	Exp(B) = 1.044	Exp(B) = 1.048	NRA	Exp(B) = 0.371	**Exp(B) 0.163**	**Exp(B) = 0.222**
	*p* = 0.720	*p* = 0.720	*p* = 0.711		*p* = 0.119	***p* = 0.019**	***p* = 0.037**
DBO Blur	**Exp(B) = 0.810**	Exp(B) = 0.908	Exp(B) = 0.877	PRA	**Exp(B) = 3.443**	Exp(B) = 1.994	**Exp(B) = 8.570**
	***p* = 0.034**	*p* = 0.236	*p* = 0.126		***p* = 0.047**	*p* = 0.206	***p* = 0.006**
DBO Break	Exp(B) = 0.941	Exp(B) = 1.069	Exp(B) = 0.902	AC/A (plus)	Exp(B) = 0.935	**Exp(B) = 0.717**	Exp(B) = 1.193
	*p* = 0.224	*p* = 0.191	*p* = 0.070		*p* = 0.551	***p* = 0.026**	*p* = 0.141
DBO Recovery	Exp(B) = 1.000	Exp(B) = 1.041	Exp(B) = 0.960	AC/A (minus)	**Exp(B) = 1.359**	Exp(B) = 0.936	Exp(B) = 1.065
	*p* = 1.000	*p* = 0.497	*p* = 0.482		***p* = 0.041**	*p* = 0.563	*p* = 0.581
NLP	**Exp(B) = 0.868**	Exp(B) = 0.919	Exp(B) = 0.989	MAF	**Exp(B) = 0.606**	**Exp(B) = 0.345**	Exp(B) = 0.817
	***p* = 0.031**	*p* = 0.129	*p* = 0.829		***p* = 0.048**	***p* = 0.014**	*p* = 0.326
NBI Blur	Exp(B) = 0.963	**Exp(B) = 0.775**	Exp(B) = 1.750	BAF	*p* = 0.166	**Exp(B) = 0.472**	Exp(B) = 0.857
	*p* = 0.554	***p* = 0.011**	*p* = 0.122		Exp(B) = 0.734	***p* = 0.018**	*p* = 0.487
NBI Break	Exp(B) = 0.964	Exp(B) = 0.970	Exp(B) = 1.096	Stereopsis	Exp(B) = 1.020	Exp(B) = 1.017	**Exp(B) = 1.075**
	*p* = 0.574	*p* = 0.639	*p* = 0.185		*p* = 0.077	*p* = 0.133	***p* = 0.017**
NBI Recovery	Exp(B) = 1.038	Exp(B) = 1.025	Exp(B) = 1.199	NPC	Exp(B) = 1.093	**Exp(B) = 1.876**	Exp(B) = 1.455
	*p* = 0.712	*p* = 0.810	*p* = 0.127		*p* = 0.624	***p* = 0.014**	*p* = 0.080
NBO Blur	Exp(B) = 0.883	**Exp(B) = 0.827**	Exp(B) = 1.750	NPA	**Exp(B) = 0.456**	Exp(B) = 0.993	**Exp(B) = 0.436**
	*p* = 0.104	***p* = 0.038**	*p* = 0.122		***p* = 0.018**	*p* = 0.980	***p* = 0.023**

Significant values are in [bold].

## Discussion

In an era of growing technology usage, the demand for binocular vision is changing, resulting in the emergence of conditions such as CVS. Patients with CVS caused by technological products with user interfaces different from conventional electronic devices include: Virtual Reality headsets or wearable devices, Augmented Reality glasses, Brain-Computer Interface, and Virtual assistants^28,29^. According to the analysis in this study, the values of binocular visual function among Taiwan high-tech industrial engineers^26^ were significantly different from the standard values and Taiwan Norms, and the actual values were mostly lower than the standard values. Previous studies indicated that technological developments^30^ are one of the main factors that change the visual function; in addition, the overuse of near vision as well as excessive use of accommodation and cohesion, resulting in functional fatigue or rigidity, may lead to poor overall binocular visual performance^31–33^.

In addition, we speculated that lifestyle habits and visual demands are the primary factors influencing these results. The work of high-tech industry employees mainly involves the use of electronic devices, which leads to significantly longer periods of near-distance visual tasks compared to the general population. The impact of computer usage time^34^ is greater than previously reported influences such as gender^35^, age^36^, and refractive error^37^. Furthermore, the Morgan and Scheiman OEP standards have been established for 80 years. Over this period, refractive issues in humans might also contribute to the deterioration of binocular visual function^7^. Apart from this, the multifunctionality of electronic products, such as tablet magnification and enhanced brightness, combined with the customization of reading glasses, might reduce the necessity for such high binocular visual standards to accomplish tasks^38–40^.

The average risk level of the CVSS-17 total score and ISF factors indicates a higher-than-normal risk for Taiwanese high-tech industry participants. This suggests that these subjects frequently experience noticeable discomfort in their eyes. Among the self-perceived reactions, eye fatigue, feeling burdened, increased blink frequency, and seeing double images are particularly prominent. These reactions are often correlated with individual characteristics, such as lag of accommodation^41,42^, micro-fluctuations in accommodation^43^, or asthenopia^27^. However, the average risk level for ESF factors is moderate, indicating that the discomfort caused by ESF had a relatively small impact on the study subjects, and might be related to the lighting and working distance^44,45^.

The limitation of this study was the sample size and the amplitude of accommodation of a twenty-year-old person is completely different from a forty-year-old person, this issue can affect the nature of binocular vision parameters evaluated at different ages. In future research, it would be beneficial to include a more diverse pool of participants, and conducting analyses grouped by age might hold greater significance for clinical implications.

## Conclusion

The study found that Taiwanese high-tech workers have evolved from simple accommodative anomalies to more complex binocular visual stress involving both accommodative and convergence anomalies. It is suggested that regular check-ups, appropriate rest breaks, appropriate visual correction or prescribing occupational progressive lenses along with visual training might alleviate stress and improve symptoms of visual fatigue and binocular abnormalities. Moreover, proper ergonomic desk and chair configuration, are also trends that can enhance the efficiency of high-tech industry employees.

Future research directions could explore whether high-tech workers in other countries or regions experience similar visual developments or possess more advanced strategies or aids for symptom relief compared to those in Taiwan. Increasing the sample size in subsequent studies can enhance the evaluation of CVS symptoms and major binocular abnormality items through the CVSS-17 questionnaire, providing clinicians with references for managing CVS.

## Data Availability

All data generated or analyzed during this study are included in this published article. Correspondence and requests for materials should be addressed to C.-Y.C.
